# Homozygous *NOTCH3* p.R587C mutation in Chinese patients with CADASIL: a case report

**DOI:** 10.1186/s12883-020-01660-0

**Published:** 2020-03-02

**Authors:** Ruojie He, Huan Li, Yiming Sun, Menglong Chen, Liang Wang, Yuling Zhu, Cheng Zhang

**Affiliations:** 1 Department of Neurology, The First Affiliated Hospital, Sun Yat-sen University; Guangdong Provincial Key Laboratory of Diagnosis and Treatment of Major Neurological Diseases, National Key Clinical Department and Key Discipline of Neurology, No.58 Zhongshan Road 2, Guangzhou, 510080 China; 2 Department of Health Care, The First Affiliated Hospital, Sun Yat-sen University, No.58 Zhongshan Road 2, Guangzhou, 510080 China; 3 Department of Neurology, The First Affiliated Hospital, Jinan University, 613 W.Huangpu Avenue, Guangzhou, 510630 China

**Keywords:** CADASIL, *NOTCH3* mutation, Homozygous, Phenotypic variation

## Abstract

**Background:**

Cerebral autosomal dominant arteriopathy with subcortical infarcts and leukoencephalopathy (CADASIL) is an inherited small vessel disease caused by mutations in *NOTCH3* gene with remarkable phenotypic heterogeneity. Cases of CADASIL associated with homozygous *NOTCH3* mutations are rare and subsequently understudied. In this study, we investigate the genetic and phenotypic features within patients of CADASIL with homozygous *NOTCH3* mutations.

**Case presentation:**

We recruited two affected individuals with CADASIL from a mainland Chinese family. The proband (Patient 1), a 60-year-old male, presented with slow progressive gait instability, severe cognitive impairment, and emotional disorder for more than 2 years with a history of ischemic stroke and hypertension. His younger brother (Patient 2) presented with apparent gait difficulties, dysarthria as well as cognitive decline at 59 years old. Brain magnetic resonance imaging (MRI) showed diffused white matter lesions involving bilateral periventricular white matter, semioval center region, and anterior temporal lobes. Molecular genetic testing identified a homozygous variant, c.1759C > T (p.R587C), in *NOTCH3* gene in both patients. Pathological analysis revealed granular osmiophilic material (GOM) deposits in small arterial walls of skin from the proband. The diagnosis of CADASIL was confirmed.

**Conclusions:**

Our cases of CADASIL with homozygous mutation c.1759C > T (p.R587C) in *NOTCH3* share similar manifestation to the patients with heterozygous same mutation reported previously. Other than genetic factors, vascular risk factors or environmental factors might contribute to the phenotypic variation of CADASIL.

## Background

Cerebral autosomal dominant arteriopathy with subcortical infarcts and leukoencephalopathy (CADASIL) (OMIM: 125310) is an autosomal dominant small vessel disease predominantly caused by mutations in *NOTCH3* gene (HGNC ID: 7883), which encodes internal, external and transmembrane domains of NOTCH3 protein. The extracellular domain consists of 34 epidermal growth factor-like repeats (EGFR), each including 6 cysteine residues. Most identified mutations localize in *NOTCH3* result in an odd number of cysteine residues within a given EGFR. The clinical presentations include recurrent subcortical ischemic events, cognitive impairment or vascular dementia, migraine, psychiatric disorders as well as diffuse white matter lesions and lacunar infarcts in brain magnetic resonance imaging (MRI) [1, 2]. Systemically, patients develop pathognomonic granular osmiophilic material (GOM) deposits in the vessel walls, which could be detected in skin biopsies using electron microscopy [3].

Since the first identification of *NOTCH3* mutations in 1996 [4], over 200 mutations have been identified [5]. However, few cases homozygous for a *NOTCH3* mutation have been reported to date [6–12], and the specific phenotype-genotype spectrum of patients with homozygous *NOTCH3* mutations has not been delineated. In addition, it remains to be determined whether the homozygosity of *NOTCH3* mutation is responsible for more severe phenotype in patients of CADASIL, and existing reports offer contradictory observations [8, 10].

Here, we report the first patients of CADASIL from a Chinese family with homozygous *NOTCH3* mutation c.1759C > T (p.R587C), and pathological analysis confirm GOM deposits in the vessel walls in proband patient.

## Case presentation

### Patient 1

**Fig. 1:**
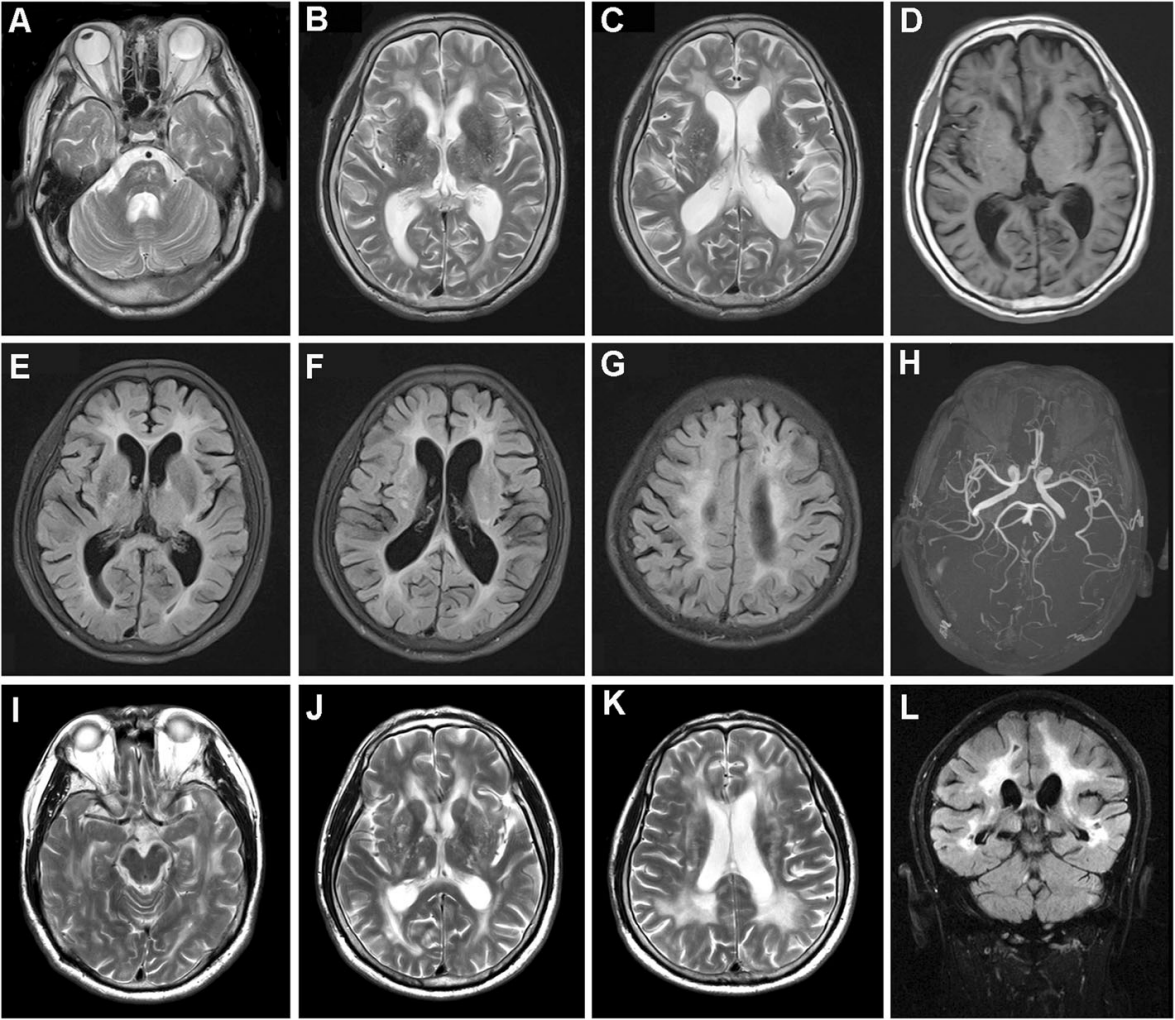
Brain MRI of the patient. Brain MRI of the patient 1 (**a-h**) and patient 2 (**i-l**) in the family with CADASIL showed diffused white matter hyperintensities as well as lacunar infarcts on T1-weighted, T2-weighted and FLAIR imaging

The proband, a 60-year-old man, suffered from progressive gait instability particularly when walking up and down stairs, cognitive decline, and emotional disorder for more than 2 years. At the age of 47 years, he experienced an ischemic stroke and had mild disability in his left limbs. In the following 10 years, left side hemiparesis did not progress. There was no history of migraine and dizziness. He had hypertension for more than two decades and blood pressure was not well controlled. Neurological examination revealed impaired higher cortical function, gait ataxia, positive Romberg’s sign, the presence of bilateral Rossolimo’s signs, and palm-chin reflexes. Decreased muscle strength and brisk tendon reflexes were found on the bilateral lower limbs. The neuropsychological evaluation showed considerable cognitive decline including impairments of memory and language ability, with mood disturbances manifesting as both apathy and depression. His performance on the mini-mental status examination (MMSE) showed a moderate cognitive impairment (a score of 20 out of 30). However, he scored 14 out of 30 on the Montreal Cognitive Assessment (MOCA), indicating severe cognitive impairment. Neck vessel doppler sonography revealed left carotid atherosclerosis and the echocardiography evaluation showed hypertrophy of the ventricular septum and enlargement of the left atrium. Brain MRI on T2-weighted and fluid attenuation inversion recovery (FLAIR) imaging revealed diffused white matter hyperintensities mostly involving the bilateral periventricular white matter, semioval center, and left anterior temporal lobe in association with multiple lacunar infarcts predominantly located in subcortical white matter, brainstem, and basal ganglia (Fig. 1a-c, e-g). The small lacunar infarcts could be readily observed on T1-weighted imaging with decreased signal intensity (Fig. 1d). Cerebral magnetic resonance angiography presented stenosis of the intracranial arteries with cerebral arteriosclerosis (Fig. 1h). Based on clinical and neuroimaging findings, he was clinically suspected of having the diagnosis of CADASIL and underwent genetic and pathological analyses.

**Fig. 2:**
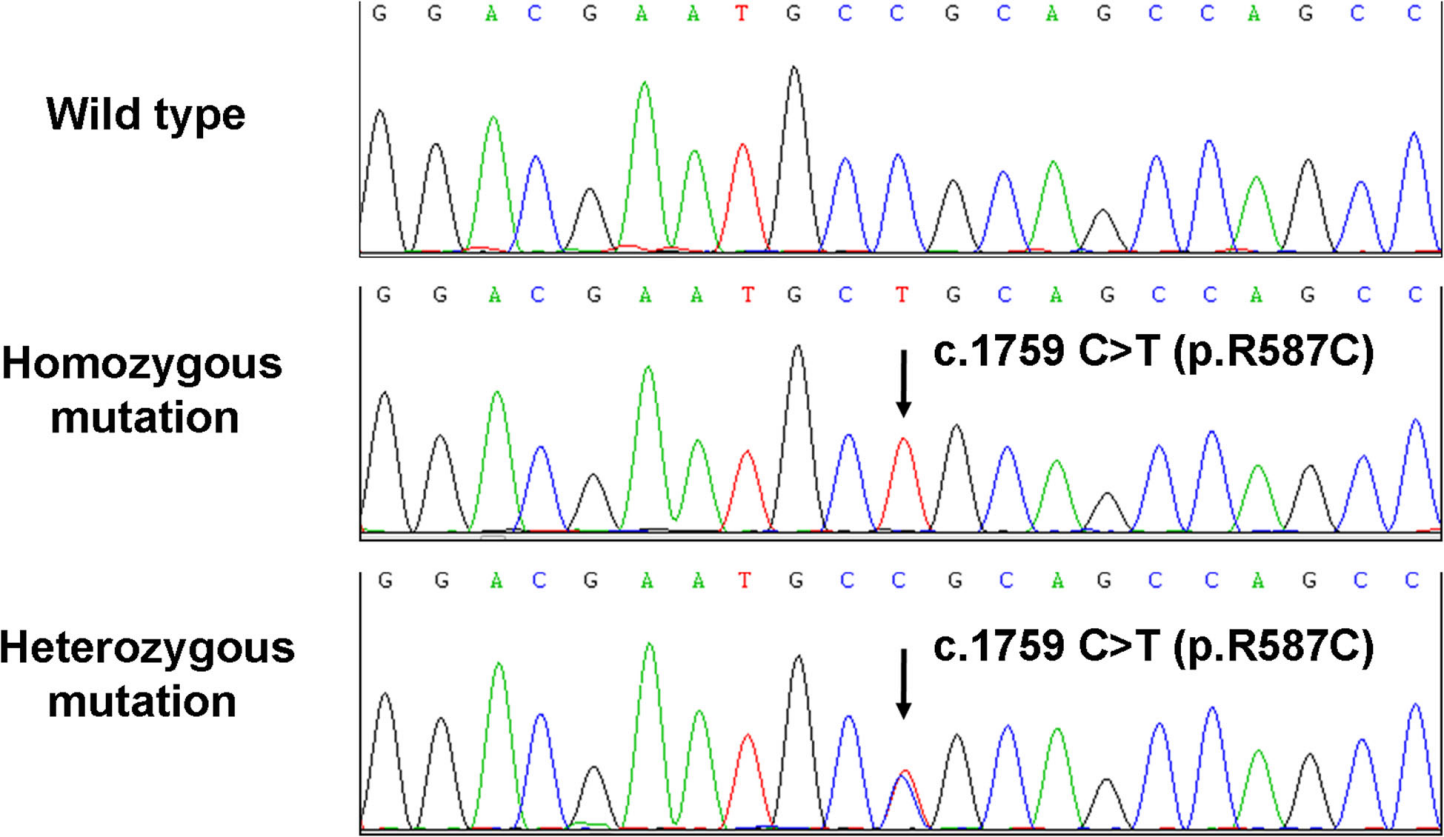
DNA Sequencing analysis of *NOTCH3* in three family members with CADASIL. The homozygous *NOTCH3* c.1759C > T (p.R587C) mutation in exon 11 was detected in the patient 1 and patient 2, while the same heterozygous mutation was detected in the son of patient 1

**Fig. 3:**
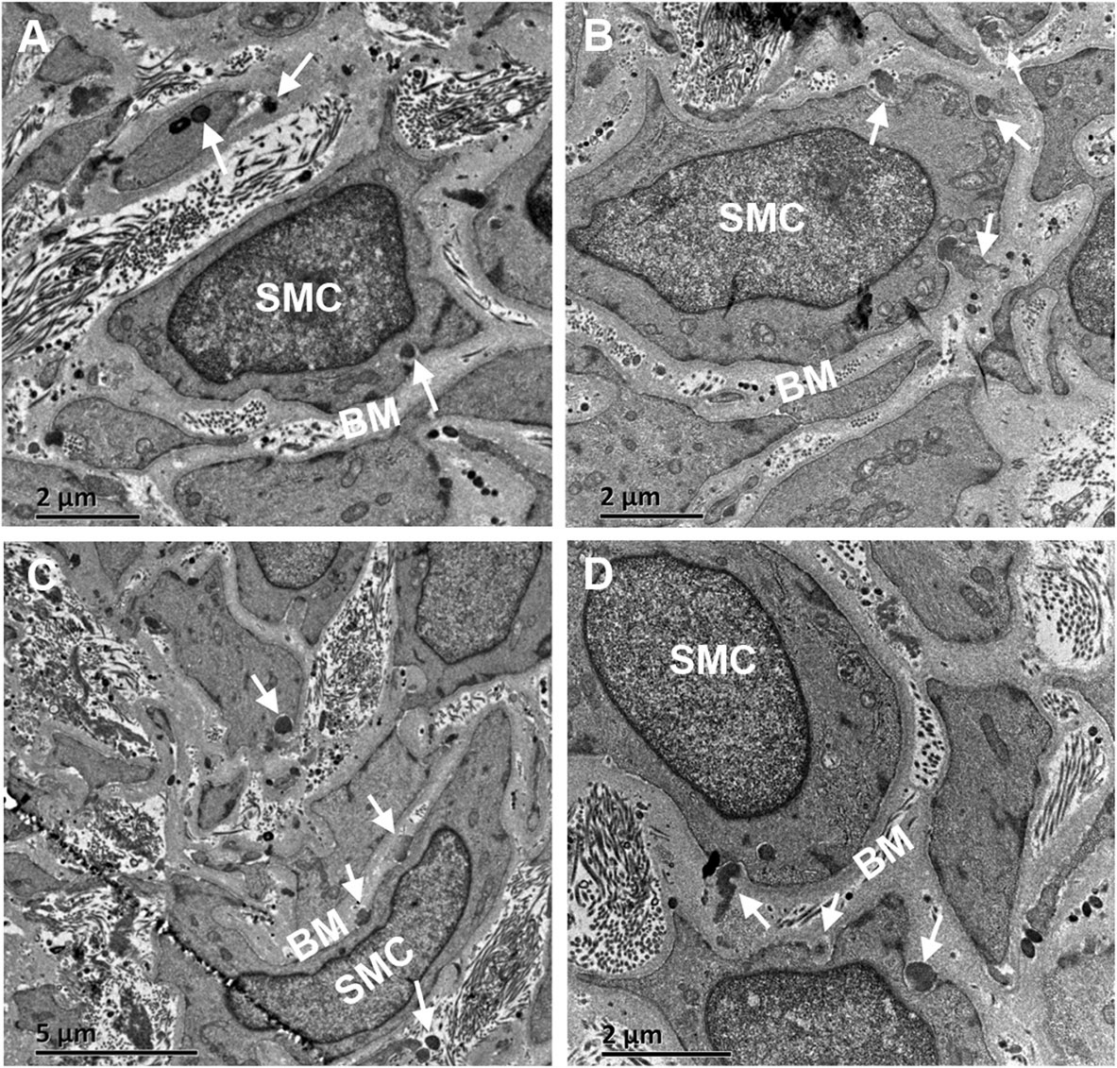
Ultrastructural analysis using Transmission electron microscopy (TEM) of small dermal arteries from skin biopsy of the proband. GOM deposits (white arrows) showing different shapes were mainly located between vascular SMC and the basement membrane (BM). **a, b** TEM magnification × 8000. **c** TEM magnification × 5000. **d** TEM magnification × 10,000

Written informed consent was obtained from the patients and the study was approved by the Institutional Review Board of The First Affiliated Hospital of Sun Yat-sen University. The direct sequencing of the *NOTCH3* gene identified the homozygous c.1759C > T (p.R587C) missense mutation in exon 11 (Fig. 2), which was identified in dbSNP (rs754554486) and Human Gene Mutation Database (HGMD) (CM061879) with a gnomAD allele frequency of 3.616e-5. This change was predicted as damaging variant based on analysis using PolyPhen-2 software. Furthermore, the patient underwent skin biopsy on his right lower limb and electron microscopic analysis revealed GOM deposits between vascular smooth muscle cells (SMCs) and the basement membrane, and occasionally within SMCs in small arterial walls (Fig. 3).

### Patient 2

The younger brother of the proband was a 59-year-old man presenting with apparent gait difficulties and clumsiness of movement from the age of 57. He gradually developed slurred speech and mild weakness of the bilateral upper limbs, and had distinct difficulty in completing routine work. He had a transient ischemic attack (TIA) at the age of 50 and recovered. There was also no history of migraine. No vascular risk factors were found. The neurological examination revealed gait ataxia, dysarthria, reduced mild power on bilateral upper limbs as well as brisk deep tendon reflexes in all limbs. The bilateral Rossolimo’s signs and right palm-chin reflex were positive. Neuropsychological examination showed impairment of linguistic and calculative functions and mood disturbance like apathy. Brain MRI findings demonstrated confluent and symmetrical distribution of white matter lesions in bilateral periventricular white matter, semioval center region, and anterior temporal lobes on T2-weighted and FLAIR imaging. Multiple lacunar infarcts were detected predominantly in basal ganglia, thalamus, brainstem, and subcortical white matter (Fig. 1i-l). His MMSE score was 23 out of 30, indicating a mild cognitive impairment. The same homozygous *NOTCH3* c.1759C > T (p.R587C) mutation was detected (Fig. 2).

The proband’s father had a history of ischemic stroke and died at the age of 50, and the proband’s mother died at the age of 69 because of heart disease. Unfortunately, no further clinical information from either individual was available. The proband’s son, a 34-year-old man, was clinically asymptomatic and his neuroimaging finding was normal. Nevertheless, the heterozygous *NOTCH3* c.1759C > T (p.R587C) mutation was detected (Fig. 2).

## Discussion and conclusions

We reported the homozygous missense c.1759C > T (p.R587C) mutation in *NOTCH3* gene discovered in two brothers from a mainland Chinese family with CADASIL confirmed pathologically. Clinically, both patients in this study presented with ischemic stroke, gait instability, cognitive impairment, and psychiatric disorders which were highly compatible with the phenotypic spectrum of CADASIL in previous studies from both Chinese and other ethnic backgrounds [13–16]. The confluent white matter hyperintensities as well as multiple lacunar infarcts shown on brain MRI scanning of our patients were in accordance with typical neuroimaging features of CADASIL. None of the patients in present study reported a history of migraine with or without aura, which is consistent with a relatively low frequency of migraine in patients of CADASIL from Asian countries [16, 17]. The diagnosis of CADASIL was genetically confirmed by the detection of a homozygous mutation in exon 11 of *NOTCH3* gene, which was predicted to cause a substitution of arginine with cysteine (p.R587C) in the encoded receptor. The ultrastructural examination of dermal arteries performed via skin biopsy from the proband showed pathognomonic GOM deposits around irregularly shaped SMCs. In addition, the proband’s son, a heterozygous carrier of *NOTCH3* c.1759C > T (p.R587C) mutation, was asymptomatic likely due to his young age. Though clinical and genetic assessment were unavailable from the proband’s parents, we posited that they were likely to be carrying the heterozygous *NOTCH3* c.1759C > T (p.R587C) mutation due to the homozygosity in the proband and his younger brother.

To our knowledge, the homozygous *NOTCH3* c.1759C > T (p.R587C) mutation has not been reported before, though the heterozygous cases were discussed in previous studies [15, 18, 19]. Whether homozygous *NOTCH3* mutations are associated with much more severe phenotypes in patients with CADASIL remains to be controversial. Some patients with homozygous *NOTCH3* mutations exhibit more aggressive clinical or pathological manifestations when compared to their heterozygous family members or age-matched patients with heterozygous mutations [7, 9, 10], whereas others exhibit similar phenotypes with heterozygous patients [6, 8]. In a study from Taiwan, a proband with homozygous c.1630C > T (p.R544C) mutation in *NOTCH3* gene presented with a later age of onset and mildly increased clinical severity compared to her heterozygous sister [9]. In another report, a 65-year-old patient with homozygous *NOTCH3* c.1732C > T (p.R578C) mutation had parallel phenotypes to those who harbored heterozygous one [6]. In a recent study, a case of CADASIL from Thailand with a homozygous c.1672C > T mutation in *NOTCH3* gene presented with the largest number of cerebral microbleeds ever recorded, probably caused by a homozygous state and uncontrolled vascular risk factors [20]. In present study, clinical manifestations including ischemic stroke, motor disability, cognitive impairment, mood disturbance as well as lack of migraine in our patients with homozygous *NOTCH3* c.1759C > T (p.R587C) mutation were accordant with those described in a Korean patient who was heterozygous for the same mutation. Moreover, the diffused white matter lesions on brain MRI examination and GOM deposits in dermal arterioles were comparable between our patients and the Korean patient [18]. These observations suggest that the homozygosity of *NOTCH3* c.1759C > T (p.R587C) mutation are probably not associated with more severe phenotypes of CADASIL.

Previous studies indicated that the identical *NOTCH3* gene mutation may result in considerable phenotypic variability across unrelated patients, and even members within the same family, suggesting that additional genetic factors, environmental cues or other vascular risk factors are likely to influence the disease progression [9]. Intriguingly, the proband patient and his younger brother in this study displayed mild variation on clinical phenotype though carrying the identical *NOTCH3* gene mutation and from the same family. The onset of age for the first ischemic stroke was earlier in the proband when compared to his younger brother. Accordingly, the proband had vascular risk factors like hypertension and left carotid atherosclerosis whereas his younger brother had no vascular risk factors, which indicated the existence of modifying factors other than sole *NOTCH3* gene mutation on disease progression and phenotypic variability, as described in previous reports.

In conclusion, we present two cases of CADASIL from a mainland Chinese family with homozygous *NOTCH3* c.1759C > T (p.R587C) mutation, one of them is confirmed pathologically. The clinical presentations of our patients with homozygous *NOTCH3* c.1759C > T (p.R587C) mutation are in accordance with that of patients with heterozygous the same mutation described before. Vascular risk factors, environmental factors, and additional unknown genetic factors might contribute to phenotypical heterogeneity of CADASIL.

## Data Availability

The authors declare that all the data are contained within the manuscript.

## Abbreviations

CADASIL: Cerebral autosomal dominant arteriopathy with subcortical infarcts and leukoencephalopathy
EGFR: Epidermal growth factor-like repeats
FLAIR: Fluid attenuation inversion recovery
GOM: Granular osmiophilic material
HGMD: Human Gene Mutation Database
MMSE: Mini-mental status examination
MOCA: Montreal Cognitive Assessment
MRI: Magnetic resonance imaging
SMCs: Vascular smooth muscle cells
TIA: Transient ischemic attack
